# Maturation of Mitochondrially Targeted Prx V Involves a Second Cleavage by Mitochondrial Intermediate Peptidase That Is Sensitive to Inhibition by H_2_O_2_

**DOI:** 10.3390/antiox10030346

**Published:** 2021-02-25

**Authors:** Juhyun Sim, Jiyoung Park, Hyun Ae Woo, Sue Goo Rhee

**Affiliations:** 1 National Forensic Service, 10 Ipchun-ro, Wonju 26460, Korea; jhsim86@korea.kr; 2 College of Pharmacy, Graduate School of Pharmaceutical Sciences, Ewha Womans University, Seoul 120-750, Korea; jypark89@ewhain.net; 3 College of Pharmacy, Graduate School of Applied Science and Technology for Skin Health and Aesthetics, Ewha Womans University, Seoul 120-750, Korea; 4 Yonsei Biomedical Research Institute, Yonsei University College of Medicine, Seoul 120-752, Korea; 5 Biochemistry and Biophysics Center, NHLBI, National Institutes of Health, Bethesda, MD 20892, USA

**Keywords:** peroxiredoxin V, mitochondria targeting sequence, mitochondrial intermediate peptidase, hydrogen peroxide

## Abstract

Prx V mRNA contains two in-frame AUG codons, producing a long (L-Prx V) and short form of Prx V (S-Prx V), and mouse L-Prx V is expressed as a precursor protein containing a 49-amino acid N-terminal mitochondria targeting sequence. Here, we show that the N-terminal 41-residue sequence of L-Prx V is cleaved by mitochondrial processing peptidase (MPP) in the mitochondrial matrix to produce an intermediate Prx V (I-Prx V) with a destabilizing phenylalanine at its N-terminus, and further, that the next 8-residue sequence is cleaved by mitochondrial intermediate peptidase (MIP) to convert I-Prx V to a stabilized mature form that is identical to S-Prx V. Further, we show that when mitochondrial H_2_O_2_ levels are increased in HeLa cells using rotenone, in several mouse tissues by deleting Prx III, and in the adrenal gland by deleting Srx or by exposing mice to immobilized stress, I-Prx V accumulates transiently and mature S-Prx V levels decrease in mitochondria over time. These findings support the view that MIP is inhibited by H_2_O_2_, resulting in the accumulation and subsequent degradation of I-Prx V, identifying a role for redox mediated regulation of Prx V proteolytic maturation and expression in mitochondria.

## 1. Introduction

Peroxiredoxins (Prxs) are a large family of peroxidases found in all forms of oxygen-dependent life, from bacteria to mammals, that reduce peroxides such as hydrogen peroxide, alkyl hydroperoxides, and peroxynitrite [1]. The peroxidase reaction depends on a reactive cysteine residue in their active sites that is conserved among all Prxs [2] [3]. This catalytic Cys is referred to as the peroxidatic Cys (C_P_) to reflect its sensitivity to oxidation by peroxides [4]. In addition to C_P_, most Prxs contain another conserved cysteine designated the resolving Cys (C_R_). On the basis of the location or absence of the C_R_ residue, Prxs are classified into typical 2-Cys, atypical 2-Cys, and 1-Cys Prx subfamilies [3]. Mammalian cells express six isoforms of Prx: four 2-Cys Prx isoforms (Prx I to IV), one atypical 2-Cys Prx isoform (Prx V), and one 1-Cys Prx isoform (Prx VI). Typical 2-Cys Prxs are obligate homodimers and form an intermolecular disulfide bond between C_P_ and C_R_ following the oxidation of C_P_-SH to sulfenic form (C_P_-SOH) by peroxides and subsequent reaction of C_P_-SOH with C_R_-SH. The disulfide linkage is reduced by thioredoxin (Trx) completing a catalytic cycle. Because the disulfide formation between C_P_-SOH and C_R_-SH is a slow process, the C_P_-SOH of 2-Cys Prxs often undergoes further oxidation to sulfinic acid (C_P_-SO_2_H) during catalysis, resulting in the inactivation of peroxidase activity [1,5]. The hyperoxidized 2-Cys Prxs can be reactivated through an ATP-consuming reduction reaction catalyzed by sulfiredoxin (Srx) [6,7].

Prx V, also named PMP20 and AOEB1166 before its peroxidase activity was demonstrated, is the only mammalian atypical 2-Cys Prx [8,9,10]. Contrary to typical 2-Cys Prxs, which form an intermolecular disulfide bond, Prx V forms an intramolecular disulfide bond between C_P_ (Cys^48^) and C_R_ (Cys^152^) during a catalytic cycle [8,10,11].

Prx V is found in the cytosol, mitochondria, nucleus, and peroxisomes, whereas Prx I, II, and VI are localized mainly in the cytosol, Prx III is restricted to mitochondria, and Prx IV is predominantly in the endoplasmic reticulum (ER) [3,10]. The wide subcellular distribution of Prx V is attributable to the presence of two translation initiation sites in Prx V mRNA and two subcellular localization signals in their translated products [8,12]. Translation from the first AUG produces a 209-amino acid residue mouse protein, whereas the use of the second AUG produces a 161-amino acid residue protein (both residue numbers are after N-terminal demethionylation). Both the long (L-Prx V) and short (S-Prx V) forms of Prx V contain a COOH-terminal SQL peroxisomal-targeting sequence, whereas the L-Prx V contains a 49-amino acid N-terminal mitochondrial targeting sequence (MTS) [10].

The maturation of most matrix precursor proteins is achieved through a single cleavage of the presequence by the mitochondrial processing peptidase (MPP) located in the mitochondrial matrix [13,14]. Some matrix preproteins undergo a second proteolysis by the mitochondrial intermediate peptidase (MIP) after MPP cleavage [13,14,15,16]. Earlier sequence alignment of eight MIP substrates (five from *Saccharomyces cerevisiae*, and one each from *Neurospora crassa*, mouse, and human) revealed a cleavage site motif, the R-10 motif, with a consensus sequence comprising an Arg in position −10 (position relative to the mature protein), hydrophobic residues (Phe for six substrates, and Leu or Ile for one substrate each) in position −8, and small uncharged residues (Ser for four substrates, Thr for three, and Gly for one) in position −5: R-X↑(F/L/I)-XX-(S/T/G)-XXXX↑X (where the first arrow indicates cleavage by MPP and the second arrow represents cleavage by MIP) [13,17]. As MIP removes an octapeptide from the N-terminus of the preprotein intermediate generated by MPP, MIP is also called octapeptidyl aminopeptidase 1 (Oct1).

Later global analysis of mature mitochondrial N-termini in yeast by mass spectrometry identified a total of 14 Oct1 substrates including Prx 1, one of the five Prx isoforms in yeast that is exclusively located in the mitochondria [18,19]. Inspection of the cleavage motif of the 14 Oct1 substrate proteins revealed that the amino acids in positions -10 and -5 are rather variable, whereas all 14 substrates contained a hydrophobic residue (Phe for 11, Leu for 2, or Ile for 1 substrate) in position −8 [18].

Sequential cleavage has been proposed to have various functions, including the provision of a compatible MIP site, regulation of submitochondrial sorting, protein complex assembly, and protein stabilization [14,15,20]. Among the 17 known MIP substrates (14 *S. cerevisiae*, 1 *N. crassa*, 1 mouse, and 1 human substrate), 16 contain either Phe or Leu in position −8, which becomes the N-terminus of the intermediate protein produced after MPP cleavage [13,18]. Both Phe and Leu are primary destabilizing amino acids according to the N-end rule of protein degradation [21,22]. At the same time, cleavage of the octapeptide by MIP results in new N-termini with more stabilizing amino acids (Gln, Ser, Thr, Gly, Ala) in 14 of the 17 substrates. Thus, among the many functions proposed for this sequential processing, protein stabilization appears to be applicable to a large number of MIP substrates. Indeed, it was demonstrated that fully processed MIP substrates have a longer cellular half-life than their intermediate forms in yeast [14,18,23].

Once translocated into the mitochondrial matrix, the N-terminal 49-residue presequence of L-Prx V is proteolitically cleaved to produce a mature form that is indistinguishable from S-Prx V [10,12]. Here, we show that the maturation of L-Prx V involves a sequential cleavage by MPP and MIP. Further, we identify the intermediate Prx V (I-Prx V) as a minor band in immunoblots of mouse heart extracts and show that the conversion of I-Prx V to mature Prx V is a process inhibited by H_2_O_2_ produced in mitochondria across a range of experimental conditions.

## 2. Materials and Methods

### 2.1. Animals and Antibodies

Prx V KO mice were as described previously [24]. Srx KO mice, in which Srx is ablated specifically in steroidogenic tissues, were also described previously [25]. Prx V WT and KO mice were generated by breeding heterozygous mice. Ten- to 12-wk-old Prx V mice were used for the experiments. For other experiments 10-wk-old male C57BL/6J mice (Jackson Laboratory, Bar Harbor, ME, USA) were used. All animal experiments were performed according to the protocol approved by the Institutional Animal Care and Use committee of Ewha Womans University. The mice were housed in a temperature-controlled room (20–22 °C) with a 12 h light:12 h dark cycle. For immobilization stress, mice were exposed to stress for 0 or 1 h and euthanized after release from stress for the indicated times. Adrenal gland homogenates were then subjected to immunoblot analysis.

Rabbit antisera specific for Prx V were described previously [11]. Antibodies to GAPDH (glyceraldehyde-3-phosphate dehydrogenase) and to VDAC (Voltage-dependent anion channel) were obtained from Santa Cruz Biotechnology, Santa Cruz, CA, USA; those to Prx-SO_2_ were from Young In Frontier, Seoul, Korea; and those to Cox 4 were from Cell Signaling, Beverly, MA, USA.

### 2.2. Subcellular Fractionation

Mouse tissues were minced finely and homogenized with Douncer homogenizer (Wheaton, NJ, USA) in isolation buffer (230 mM mannitol, 70 mM sucrose, 10 mM Tris-HCl pH 7.4, 1 mM EDTA, 0.1% BSA) and centrifuged at 1000× *g* for 10 min. The supernatant was centrifuged at 12,000× *g* for 10 min. The resulting supernatant was collected as cytosol fraction, and the heavy membrane fraction (mitochondrial pellet) was washed twice with wash buffer (230 mM mannitol, 70 mM sucrose, 10 mM Tris-HCl pH 7.4) and resuspended with appropriate buffers.

### 2.3. Immunoblotting

Protein levels in HeLa cells and mouse tissues were evaluated by immunoblot analysis. Cells and tissues were lysed with cold lysis buffer (20 mM HEPES pH 7.0, 0.15 M NaCl, 10% glycerol, 1% triton X-100, 1 mM EDTA, 1 mM EGTA, 10 mM β-phosphoglycerate, 1 mM Na_3_VO_4,_ 5 mM NaF, 1 μg/mL aprotinin, 1 μg/mL leupeptin, 100 μM PMSF) using a Polytron Homogenizer or sonicator. The homogenates were centrifuged at 15,000 rpm, 4 °C for 15 min. After the protein concentration of the lysates (supernatants) was quantified using Bradford assay (Bio-Rad, Hercules, CA, USA), lysates were mixed with sample buffer (62.5 mM Tris-HCl pH 6.8, 10% glycerol, 2% sodium dodecyl sulfate, 0.0125% bromophenol blue, 2.5% β-mercaptoethanol) and heated at 95 °C for 5 min. Samples were loaded onto a sodium dodecyl sulfate-polyacrylamide gel electrophoresis (SDS-PAGE) gel and separated by electrophoresis in SDS (sodium dodecyl sulfate) buffer (3 g/L Tris, 14.35 g/L glycine, 1 g/L SDS). The proteins were transferred onto an activated polyvinylidene difluoride (PVDF) membrane with 0.45 μm pore size (Millipore, Darmstadt, Germany) by methanol with transfer buffer (3.03 g/L Tris, 14.17 g/L glycine, 20% methanol). The membrane was incubated with 5% bovine serum albumin (BSA) in tween-20 Tris-buffered saline (TTBS) at room temperature for 20 min using a rocker, followed by incubation at 4 °C overnight on a rocker with antibodies (1:2000 dilution). Immune complexes were detected with horseradish peroxidase (HRP)-conjugated secondary antibodies (Bio-Rad, Hercules, CA, USA) and enhanced with chemiluminescence reagents (Ab Frontier, Daejeon, Korea) using the LAS-3000 imager (FUJIFILM, Tokyo, Japan) or AI-600 imager (GE Healthcare, Uppsala, Sweden). The abundance of target proteins was quantitated by densitometric analysis of immunoblots. Bradford assay (SpectraMax M2 Microplate Reader, Molecular Device, San Jose, CA, USA) data were acquired at Fluorescence Core Imaging Center on Ewha Womans University.

### 2.4. Purification of Prx V Containing I-Prx V

Mouse hearts (1 g) from C57/BL6 were washed with phosphate buffered saline (PBS) and homogenized with 5 mL of buffer A (50 mM Tris-NaOH (pH 9.0), 1 mM EDTA) containing protease inhibitors. Unbroken tissues and lipids were removed by centrifugation at 25,000× *g* for 30 min. The lysate was loaded onto a DEAE sepharose column equilibrated with buffer A, and unbound flow through fraction was collected. The flow through fraction was dialyzed against buffer B (50 mM Tris-HCl (pH 8.0), 1 mM EDTA) containing 1 M ammonium sulfate and loaded onto a Phenyl sepharose column equilibrated with buffer B containing 1 M ammonium sulfate. Bound proteins were eluted with a decreasing gradient of ammonium sulfate from 1 to 0.25 M. Following immunoblot analysis with anti-Prx V antibodies, fractions containing Prx V pooled, dialyzed against buffer C (50 mM sodium acetate (pH 4.5), 1 mM EDTA), and loaded onto a Mono S column equilibrated with buffer C. Proteins were eluted with a linear gradient of NaCl in buffer C with a final concentration of 1 M. Fractions containing Prx V bands including the upper minor Prx V band were pooled and dialyzed with buffer D (50 mM Tris (pH 8.0), 1 mM EDTA, 50 mM NaCl). The pooled fraction was injected onto a Blue sepharose column equilibrated with buffer D. PrxV bands were detected in the flow-through material, and those fractions containing the proteins were pooled and concentrated in an Amicon concentrator. The pooled proteins were fractionated by SDS-PAGE on 14% gel. Major and minor Prx V bands were cut out separately for mass spectral analysis.

### 2.5. RNA Isolation

The sample was lysed with 1 mL TRIzol (INVITROGEN, Carlsbad, CA, USA) using a homogenizer on ice, then 200 μL chloroform was added and vigorously vortexed for 15 s and rested for 3 min in order to separate the phenol from lysate, then centrifuged at 12,000× *g*, 4 °C for 15 min. The lysate was separated into three layers. The aqueous-top layer contained RNA and was transferred to another tube. To the tube was added 500 μL isopropanol and inverted gently four times and kept at −80 °C for 16 h for precipitation. Frozen mixture in the tube was melted on ice, then centrifuged at 12,000× *g*, 4 °C for 10 min. The pellet was washed twice with 70% ethanol in RNase free water and centrifuged at 12,000× *g*, 4 °C for 10 min, then dried. RNA pellet was resuspended with RNase free water. RNA purity and concentration were determined using Nano Drop ND-1000 spectrophotometer (DAEMYUNG, Seoul, Korea). Total RNA was isolated from cultured cells with the use of the Trizol reagent and was subjected to RT (real time) with random-hexamer primers and with the use of an ABI cDNA synthesis kit (PE Biosystems, CA, USA). The resulting cDNA was subjected to real-time PCR (Polymerase Chain Reaction) analysis with the use of SYBR Green (Perkin Elmer, Foster City, CA, USA) and an ABI PRISM 7700 system (PE Biosystems, Perkin Elmer, Foster City, CA, USA ). GAPDH was used as an internal control. The sequences of primers for mouse cDNAs (forward and reverse, respectively) are listed in Table 1.

### 2.6. Mass Spectral Sequencing of I-Prx V Peptide

The excised bands were destained in a buffer containing 100 mM NH_4_HCO_3_ (pH 8.0) and 50% (*v/v*) acetonitrile. For in-gel digestion, the destained gel pieces were dehydrated in acetonitrile and rehydrated overnight at 37 °C in 25 mM NH_4_HCO_3_ containing 20 ng/µL of sequencing-grade trypsin (Promega Co. Madison, WI, USA). Peptides were extracted with 60% (*v/v*) acetonitrile containing 0.1% trifluroacetic acid, and the extracts were evaporated to dryness in Speedvac. The resulting tryptic peptides were directly injected onto Nano UPLC-ESI-q-TOF (Ultima TM Global) (Waters Co., Milford, MA, USA). After identification of the parent ion, the mass spectrometer was set to obtain the resultant collision-induced dissociation MS/MS spectra. The internal parameters for ESI-Q-TOF were as follows: electrospray capillary voltage, 3.0 keV; cone voltage, 100 V; source temperature, 80 °C; MS survey scan, *m*/*z* 400 to 1800 with a scan time of 1 s and internal scan delay time of 0.1 s; and scan range of MS/MS acquisition, *m*/*z* 50 to 1800 with a scan time of 2 s and internal scan delay time of 0.1 s. Raw data obtained from the mass spectrometer were converted to pkl files using ProteinLynx Global Server™ (PLGS) 2.3 data processing software (Waters Co., Milford, MA, USA). MS/MS spectra were matched against amino acid sequences in SwissProt using the database search program Mascot (version 2.2.06), ProteinLynx Global SERVER (PLGS) 2.3 (Waters Co., Milford, MA, USA). All reported assignments were verified by automatic and manual interpretation of spectra using the database search program Mascot (global search engine), ProteinLynx Global SERVER (PLGS) 2.3 (Waters Co., Milford, MA, USA) and MODi (http://prix.hanyang.ac.kr/modi/ (accessed on 22 February 2021)) in a blind mode [26].

### 2.7. Cell Culture and Transfection

HeLa cells and mouse embryonic fibroblasts (MEFs) were maintained under 5% CO_2_ at 37 °C in Dulbecco’s modified Eagle’s medium supplemented with 10% fetal bovine serum, penicillin, and streptomycin. Transfection of cells with expression vectors was performed with the Neon transfection system (INVITROGEN, Carlsbad, CA, USA) and Lipofectamine 2000 (INVITROGEN, Carlsbad, CA, USA). Prx V KO MEFs were transfected with wild type or point-mutated (F42G) LPrx V with Neon transfection reagent and harvested after 24 h. For siRNA experiment, HeLa cells were transfected with control scrambled or MIP siRNA with Oligofectamine (INVITROGEN, Carlsbad, CA, USA). After 24 h, each cell was transfected again with LPrx V with Lipofectamine 2000 and harvested after 24 h. Neon, Oligofectamine, and Lipofectamine 2000 (from Invitrogen) methods were performed according to the manufacturer manual. All siRNA sequences are listed in Table 2.

### 2.8. Statistical Analysis

The Western blot protein bands were quantified via densitometry using the Image J software (IMAGEJ 1.50I, Bethesda, MD, USA). All values were expressed as means ± standard error (S.E). Statistical significance was analyzed via 2-factor ANOVA for multiple comparisons using the Graph Pad Prism software, version 6 (GRAPHPAD, Bethesda, MD, USA). A *p*-value of <0.05 was considered statistically significant.

## 3. Results

### 3.1. Determination of the N-Terminal Sequence of I-Prx V Extracted from the Minor Prx V Band

**Figure 1:**
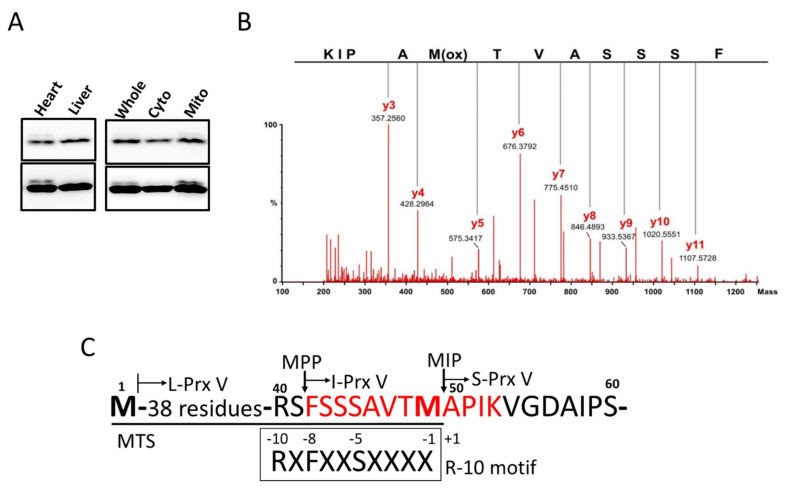
Detection of I-Prx V in mouse heart homogenate and its N-terminal amino acid sequence determined by mass spectrometry. (**A**) Mice heart and liver homogenates (left panel) and whole lysates, cytosolic, and mitochondrial fractions obtained after subcellular fractionation of heart homogenates (right panel) were subjected to immunoblot analysis with antibody to Prx V. Two different blot exposures are shown. (**B**) Collision-induced dissociation tandem mass spectrometry (MS/MS) spectrum of a peptide that is found in tryptic peptides derived from minor band (I-Prx V) but not in that derived from major band (S-Prx V). Amino acid sequence determined from the y ion series of the spectrum is indicated. (**C**) Scheme of the L-Prx V N-terminal amino acid sequence. Arrows indicate the MPP (mitochondrial processing peptidase) and MIP (mitochondrial intermediate peptidase cleavage sites. Methionines in bold type are encoded by the two in-frame AUGs used as translation-initiation sites. Amino acid sequence of I-Prx V tryptic peptide determined by mass spectrometry is highlighted in red type. MTS sequence is underlined, and R-10 motif is indicated.

Immunoblot analysis of mouse heart lysates with antibodies to Prx V revealed a major band of 17 kDa and a minor band of slightly larger size, which we designated as I-Prx V; the latter was not observed in liver lysates (Figure 1A, left panel). When the heart lysates were separated into cytosolic and heavy membrane (mitochondrial) fractions, and then subjected to immunoblot analysis, the minor upper band was detected exclusively in the mitochondrial fraction (Figure 1A, right panel). In order to identify the minor band, a fraction enriched with both the major and minor Prx V bands was obtained by subjecting mouse heart lysates to several HPLC (High-performance liquid chromatography) column chromatography steps as described in the Materials and Methods section.

Proteins in the unbound flow-through fraction from the last step were separated by SDS-PAGE (SDS-polyacrylamide gel electrophoresis gel), the two Prx V bands were identified by immunoblot analysis (not shown), the gels corresponding to the major and minor bands were excised separately, and proteins in the gels were subjected to a tryptic in-gel digestion. Peptides extracted from the gels were then analyzed by electrospray ionization-quadrupole time-of-flight (ESI-Q-TOF) mass spectrometry. A peak at *m*/*z* = 627.82, corresponding to the doubly charged ([M+2H]^2+^) ion of 1253.6 Da, was observed in the peptide sample derived from minor band but not the major band. Subsequently, collision-induced dissociation MS-MS analysis was performed on the peptide ion, as shown in Figure 1B, along with the interpretation. The y ion series of the peptide was found to be suited to the sequence KIPAM = OTVASSS (where M = O is methionine sulfoxide, likely generated during peptide preparation): y3 ion at *m*/*z* = 357.3 (356.5) for KIP; y4 ion at *m*/*z* = 428.3 (427.5) for KIPA; y5 ion at *m*/*z* = 575.3 (574.7) for KIPAM = O; y6 ion at *m*/*z* = 676.4 (675.8) for KIPAM = OT; y7 ion at *m*/*z* = 775.5 (775.0) for KIPAM = OTV; y8 ion at *m*/*z* = 846.5 (846.1) for KIPAM = OTVA; y9 ion at *m*/*z* = 933.5 (933.1) for KIPAM = OTVAS; y10 ion at *m*/*z* = 1020.6 (1020.2) for KIPAM = OTVASS; and y11 ion at *m*/*z* = 1107.6 (1107.3) for KIPAM = OTVASSS (The calculated *m*/*z* values are in parentheses).

The fact that the molecular mass of the tryptic peptide is 1253.6 Da indicates that the full sequence of the tryptic peptide is HOOC-KIPAMTVASSSF-NH_2_ (calculated mass of 1254.5 Da with a single oxygenated Met) and thus that the N-terminal residue of I-Prx V is Phe. The N-terminal sequence of L-Prx V including the 49-residue mitochondrial targeting sequence (MTS) and the MPP and MIP cleavage sites is shown in Figure 1C. The mass spectrally sequenced peptide KIPAMTVASSS corresponds to the residues from Lys in position +4 to serine in position −7 with the positions relative to the N-terminus residue Ala of mature Prx V. The residues at positions −10, −8, and −5 are Arg, Phe, and Ser, respectively. Taken together, the MTS sequence of L-Prx V closely follows the R-10 motif rule, which predicts an initial MPP cleavage that generates I-Prx V, followed by MIP cleavage that removes an octapeptide from the precursor to generate mature Prx V, which is identical to S-Prx V.

### 3.2. I-Prx V is Produced during Processing of L-Prx V to S-Prx V

**Figure 2:**
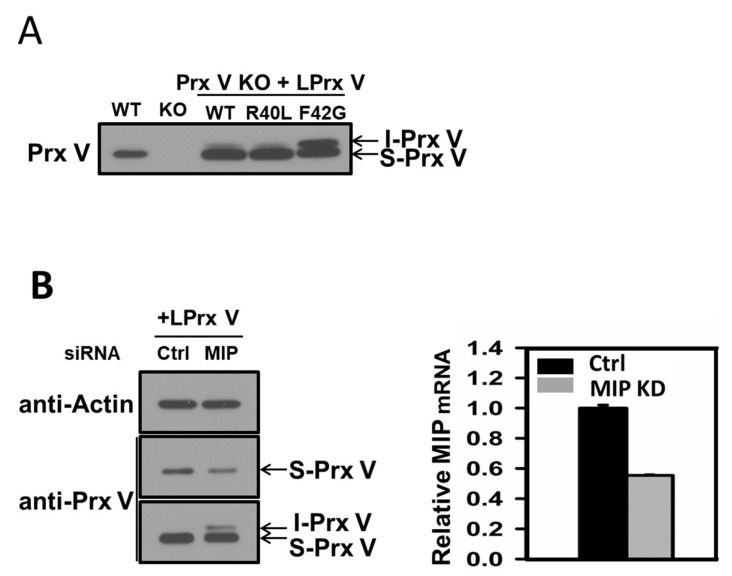
Maturation of L-Prx V involves I-Prx V that is cleaved by MIP. (**A**) Cell lysates of MEFs from wild type and Prx V KO mice as well as Prx V KO MEFs transfected with an expression vector for wild type L-Prx V, R40L L-Prx V, or F42G L-Prx V, were subjected to immunoblot analysis with antibody to Prx V. (**B**) HeLa cells were transfected with control scrambled or MIP siRNA using Oligofectamine. After 24 h, each cell was transfected again with an expression vector for LPrx V with Lipofectamine 2000 and harvested after 24 h. Cell lysates were subjected to immunoblot analysis with antibody to actin or Prx V (left panel). Two different blot exposures are shown for Prx V blot. Relative MIP mRNA levels were measured by quantitative PCR (right panel).

To test whether maturation of L-Prx V involves I-Prx V as an intermediate, mutant L-Prx V with Arg^40^ (position −10 relative to mature Prx V) replaced by Leu (R40L) or with Phe^42^ (position −8 relative to mature Prx V) replaced by Gly (F42G), along with wild-type L-Prx V, were expressed in mouse embryonic fibroblasts (Prx V KO MEFs). A minor band equivalent to I-Prx V was detected in Prx V KO MEFs expressing wild-type L-Prx V, whereas this band was not observed in MEFs from wild-type Prx V mice (Figure 2A). The intensity of the minor band was not significantly affected by mutation of Arg40 to Leu, corroborating the notion that Arg in the position −10 of the R-10 motif is not critical for processing by MPP and MIP [27]. In contrast, mutation of Phe^42^ to Gly drastically increased the minor band intensity, likely because of the replacement of the destabilizing N-terminus Phe by a stabilizing Gly residue in the intermediate protein I-Prx V. The Phe^42^ to Gly mutation appears also to partially inhibit the conversion of I-Prx V to S-Prx V by MIP (Figure 2A).

To further test MIP involvement in L-Prx V processing, HeLa cells that had been transfected with L-Prx V were subjected to MIP knock-down using siRNA. Prx V immunoblots with prolonged exposure time revealed an I-Prx V band in cells transfected with MIP-specific siRNA but not in cells transfected with scrambled siRNA (Figure 2B, left panel). It was also apparent that knock-down of MIP reduced the abundance of S-Prx V (Figure 2B, left panel). Quantitative PCR analysis showed that transfection with MIP-specific siRNA depleted MIP transcript by ~50% (Figure 2B, right panel). These results suggest that MIP mediates the conversion of I-Prx V to mature Prx V.

### 3.3. Effect of Mitochondrial ROS on the Abundance of I-Prx V and S-Prx V

**Figure 3:**
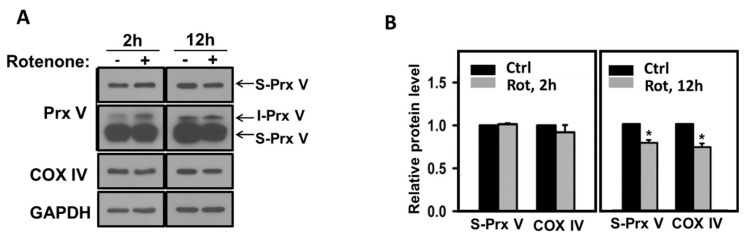
Effect of rotenone on L-Prx V maturation. HeLa cells transfected with LPrx V were treated with or without rotenone (2 μM) for 2 or 12 h and harvested. (**A**) Protein levels of Prx V, COX IV, and GAPDH in the harvested cells were measured by immunoblotting with antibodies to indicated proteins. Two different blot exposures are shown for Prx V blot. (**B**) The band intensities of S-Prx V and COX IV are normalized to the intensity of GAPDH. Data are means ± SE (*n* = 3 per group), * *p* < 0.05.

Purified mammalian MIP has previously been shown to be inactivated by thiol-blocking reagents like N-ethylmaleimide and p-mercuribenzoate and activated by dithiothreitol (DTT) [28,29], suggesting that MIP contains critical cysteine residue(s) for its catalytic activity. Critical cysteine residues of many proteins are often the target of reversible oxidation by H_2_O_2_ [30,31]. To test the effect of mitochondrially produced H_2_O_2_ on Prx V maturation, we treated L-Prx V-transfected HeLa cells with rotenone, which increases mitochondrial H_2_O_2_ production by inhibiting complex I function. We also evaluated rotenone effect on the abundance of cytochrome c oxidase subunit 4 (COX IV) as it is reportedly processed by MIP [18,32]. Though the levels of S-Prx V and mature COX IV were not affected by 2-h rotenone treatment, both levels decreased significantly by 12-h treatment (Figure 3A,B), suggesting that mitochondrial H_2_O_2_ caused a gradual inactivation of MIP likely through oxidative modification of its critical cysteine residue(s) and inhibition of the conversion of I-Prx V to S-Prx V. I-Prx V accumulated as a result of MIP inhibition would be expected to undergo degradation through the N-end rule pathway. Immunoblot intensities of I-Prx V were not strong enough to allow quantification (Figure 3A).

**Figure 4:**
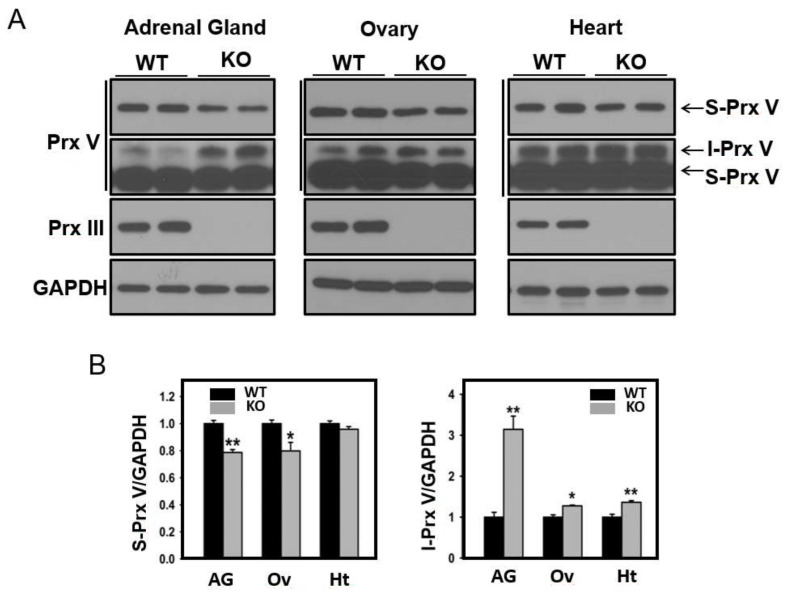
Effect of Prx III deletion on L-Prx V maturation. (**A**) Whole tissue lysates of adrenal gland, ovary, and hearts were obtained from wild type and Prx III KO mice were subjected to immunoblot analysis with antibodies to Prx V, Prx III, and GAPDH. Two different blot exposures are shown for Prx V blot. (**B**) The band intensities of S-Prx V and I-Prx V are normalized to the intensity of GAPDH. Data are means ± SE (*n* = 3 per group), * *p* < 0.05, ** *p* < 0.01.

Prx III is a strictly mitochondrial protein expressed with a MTS, and its depletion is known to increase mitochondrial H_2_O_2_. Taking advantage of the availability of Prx III-deficient mice in our laboratory [33], we evaluated the effect of Prx III ablation on Prx V processing in the adrenal gland, ovary, and heart. It was evident that Prx III ablation increased I-Prx V and decreased S-Prx in adrenal gland (Figure 4A,B). The same was true, but to a lesser extent, in ovary and heart (Figure 4A,B). Prx III ablation had no effect on Prx V transcripts in the three organs (not shown).

**Figure 5:**
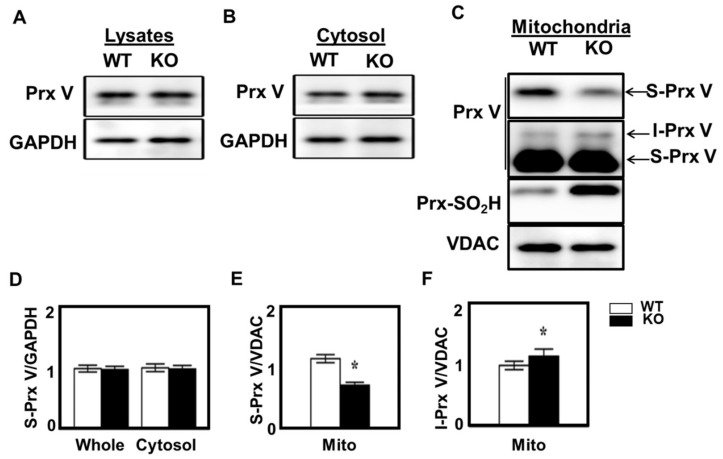
Effect of Srx deletion on L-Prx V maturation in adrenal gland. Whole lysates (**A**), cytosolic (**B**), and mitochondrial (**C**) fractions obtained after subcellular fractionation of adrenal gland homogenates from wild type and Srx KO mice were subjected to immunoblot analysis with antibodies to indicated proteins. Two different blot exposures are shown for Prx V blot in (**C**). The band intensities of S-Prx V in whole lysates and cytosol are normalized to the intensity of GAPDH (**D**), and the band intensities of S-Prx V and I-Prx V in mitochondria are normalized to the intensity of VDAC (**E**,**F**). Data are means ± SE (*n* = 3 per group),* *p* < 0.05.

Prx III becomes hyperoxidized to Prx III-SO_2_H when Prx III-SOH formed during the catalytic cycle reacts with H_2_O_2_. In the absence of Srx, which reverses the hyperoxidation reaction, the catalytically inactive Prx III-SO_2_H accumulates, resulting in the elevation of mitochondrial H_2_O_2_. The effect of Srx ablation on Prx V processing in adrenal gland was studied using Srx KO mice, in which the Srx gene was deleted specifically in steroidogenic tissues [25]. Srx ablation did not affect the abundance of S-Prx V in the whole tissue lysates and cytosolic fractions of adrenal gland (Figure 5A,B,D). However, Srx ablation did reduce the abundance of Prx V and enhance the abundance of I-Prx V in mitochondrial fractions (Figure 5C,E,F).

The adrenal gland is the main organ for the production of corticosterone (CS), which is induced by the pituitary hormone adrenocorticotropic hormone (ACTH) in response to psychological or physical stress. The synthesis of CS from cholesterol is catalyzed by two NADPH-dependent cytochrome p450 reductases (CYPs) in the mitochondrial matrix. CS synthesis produces H_2_O_2_ because electron transfer processes by CYPs from NADPH to cholesterol derivatives are not perfectly coupled and are therefore leaky. Such leaked electrons react with O_2_ to produce superoxide and consequently H_2_O_2_, as is the case with electrons leaked from the mitochondrial respiratory chain [34]. During the elimination of thus-produced H_2_O_2_, the catalytic Cys-SH of Prx III undergoes hyperoxidation to Cys-SO_2_H, resulting in the accumulation of inactive Prx III-SO_2_H followed by accumulation of mitochondrial H_2_O_2_. Prx III-SO_2_H is then reduced gradually back to Prx III-SH by Srx, which is imported into the mitochondria from the cytosol in response to mitochondrially released H_2_O_2_ [25].

**Figure 6:**
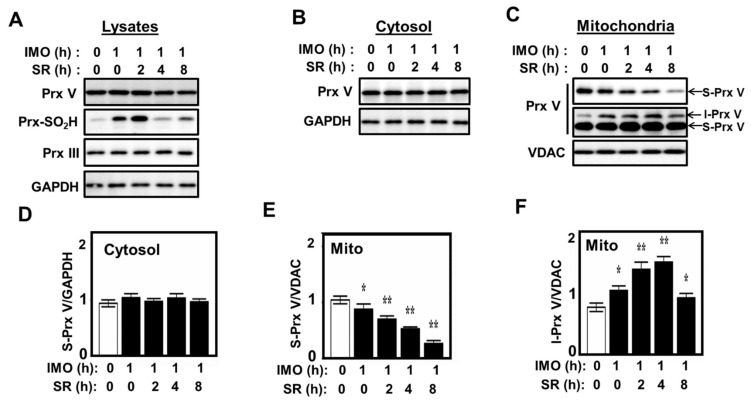
Effect of immobilization stress on L-Prx V maturation in adrenal gland. Mice were exposed to immobilization (IMO) stress for 0 or 1 h and killed after release from stress (SR) for the indicated times. Whole lysates (**A**), cytosolic (**B**), and mitochondrial (**C**) fractions obtained after subcellular fractionation of adrenal gland homogenates were then subjected to immunoblot analysis with antibodies to indicated proteins. The band intensities of S-Prx V in cytosol are normalized to the intensity of GAPDH (**D**), and the band intensities of S-Prx V and I-Prx V in mitochondria are normalized to the intensity of VDAC (**E**,**F**). Data are means ± SD from three independent experiments. * *p* < 0.05, ** *p* < 0.01.

Given that ACTH is secreted and CS is produced during physical stress [35], we examined the maturation of Prx V in the adrenal glands of mice that had been exposed to immobilization stress for 1 h and then released from the stress for the indicated times (Figure 6). Immunoblot analysis of the homogenates of adrenal glands revealed that the intensity of Prx III-SO_2_H band increased immediately by immobilization stress, with its abundance peaking 2 h after release from the stress, while the intensity of the S-Prx V band appeared to decrease slightly after release (Figure 6A). When the homogenates were separated into cytosol and mitochondrial fractions and subjected to immunoblot analysis, the intensity of the S-Prx V band in the cytosolic fractions remained constant after release (Figure 6B,D), whereas the intensity of the S-Prx V band in the corresponding mitochondrial fractions decreased steadily (Figure 6C,E). Upon longer exposure of the Prx V blot it was also possible to quantitate I-Prx V to show that immobilization stress caused an increase in the I-Prx V band intensity, which peaked 4 h after the release (Figure 6C,F). These results suggest that stress-induced steroidogenesis in the adrenal gland causes an accumulation of mitochondrial H_2_O_2_ and subsequent inhibition of MIP.

## 4. Discussion

Mitochondria contain two Prx isoforms, Prx III and Prx V, in mammalian cells. While Prx III is exclusively in the mitochondria, Prx V is also found in cytosol, nucleus, and peroxisomes. Prx V reduces peroxynitrite more effectively in the mitochondria, while Prx III is much more efficient in reducing H_2_O_2_ [10,36]. The mRNA of mouse Prx V contains two in-frame AUG initiation codons, with the first producing L-Prx V (209 amino acid residues) and the second producing S-Prx V (161 amino acid residues). S-Prx V is found in the cytosol, nucleus, and peroxisomes. L-Prx V contains a N-terminal MTS and translocates into mitochondria where its N-terminal presequence of 39 amino acid residues is proteolitically cleaved to produce a mature form that is indistinguishable from S-Prx V [10,12].

The sequential cleavage of mitochondrial Prx was previously demonstrated with yeast Prx 1, which is exclusively located in the mitochondria [18,19]. The study on yeast Prx 1 revealed that Prx 1 is initially cleaved by MPP to generate an intermediate protein with the destabilizing Phe at its N-terminus and that Oct 1(also known as MIP) removes the next eight amino acids from the N-terminus to convert the destabilized intermediate to mature Prx 1 with a stabilizing N-terminal Lys [19,23]. In this study, we show that the maturation of mouse L-Prx V also involves the two-step cleavage by MPP and MIP; that the intermediate I-Prx V has a destabilizing Phe at its N-terminus; and that removal of an octapeptide from the N-terminus of I-Prx V by MIP converts unstable I-Prx V into stable mature S-Prx V with a stabilizing N-terminalAla. Sequential cleavage of human L-Prx V by MPP and MIP are predicted to produce proteins with Phe and Ala, respectively, at their N-termini based on amino acid sequence reported in (10).

Though the I-Prx V band was not detectable in immunoblots of HeLa cells, it became apparent as a minor band when MIP activity was reduced by partial knock-down of MIP. MIP knock-down also caused a decrease in the amount of S-Prx V, suggesting that as the conversion of I-Prx V to S-Prx V is slowed, more I-Prx V is degraded according to the N-end rule pathway of protein degradation [21,22]. Increased I-Prx V and decreased S-Prx V were also observed when mitochondrial H_2_O_2_ was increased with rotenone. This is consistent with the notion that MIP contains critical Cys residue(s) for its catalytic activity that are sensitive to oxidation by H_2_O_2_. Though the sensitivity of MIP activity to thiol-blocking reagents has been known for a long time [28,29], the sensitive Cys residues were not known until recently, when comprehensive and quantitative mapping of the mouse cysteine proteome using noble cysteine-reactive phosphate tag revealed that Cys279 and Cys403 of MIP are sensitive to oxidation by ROS in vivo [37].

I-Prx V was detectable as a minor band in immunoblots of whole cell extracts of mouse tissues including heart, ovary, and adrenal gland. In those tissues in Prx III KO mice, where mitochondrial H_2_O_2_ levels are enhanced, increased I-Prx V and decreased S-Prx V were observed, most prominently in the adrenal gland. As expected, I-Prx V was found in the mitochondrial but not cytosolic fraction. The adrenal gland is an organ where physical stress induces corticosterone steroidogenesis and consequently H_2_O_2_ production, followed by a slow conversion of active Prx III-SH to inactive Prx III-SO_2_H. Immobilization stress for 1 hr resulted in an increase in the amount of I-Prx V that peaked at 4 hr and diminished thereafter, as well as a steady decrease in the amount of S-Prx V. Furthermore we previously showed that mitochondrial S-Prx V in kidney was significantly reduced when mice were subjected to renal ischemia/reperfusion injury, which is accompanied by mitochondrial ROS production [24]. Together, these in vivo experiments support the notion that MIP is inhibited by mitochondrially produced H_2_O_2_.

Currently Cox4 and Prx V are the only known substrates of MIP in mammalian cells. Oct1 substrates identified in yeast are mostly subunits of electron transport chain complexes such as Sdh4 (succinate dehydrogenase, subunit of respiratory chain complex II), Rip1 (Rieske iron-sulfur-protein, subunit of respiratory chain complex III), Cox4 (cytochrome c oxidase, subunit 4 of respiratory chain complex IV), the citrate cycle enzyme Mdh1 (mitochondrial malate dehydrogenase) [38], or the H_2_O_2_-metabolizing enzyme Prx1 [19]. MIP and Oct 1 are highly conserved and the targets of MIP/Oct1 also appear to be evolutionarily conserved as evidenced by the cleavage of COX IV and 1-Cys Prx member (Prx 1 and Prx V) by MIP/Oct1. Since most of these MIP/Oct1 substrate proteins either directly or indirectly influence mitochondrial respiratory chain activity, it is likely that changes in MIP/Oct1 activity affect mitochondrial bioenergetics. Electron transfer through the respiratory chain and nutrient oxidation also produces H_2_O_2_. Inhibition of MIP by H_2_O_2_ thus likely constitutes a feedback loop coupling H_2_O_2_ generation to ATP production. It is interesting to note that recessive mutations in the human MIP gene cause a wide variety of syndromes including left ventricular non-compaction, development delay, hypotonia, seizures, cataracts, and early childhood death [38].

## 5. Conclusions

Our results indicate that maturation of L-Prx V in mitochondria involves sequential cleavage by MPP and MIP and that the MIP-mediated step is inhibited by H_2_O_2_ likely because H_2_O_2_ inhibits MIP activity by oxidizing the unknown cysteine residue(s) of MIP.

## Figures and Tables

**Table 1:** Sequence of qPCR(quantitative real time PCR) primers.

Target Gene	Forward Primer (5′-3′)	Reverse Primer (5′-3′)
GAPDH	AGAACATCATCCCTGCATCC	GGTCCTCAGTGTAGCCCAAG
LPrx V	AGAAGCAGGTTGGGAGTGTG	CTTTCTTGCCCTTGAACAGC
SPrx V	GGCATTTACACCTGGCTGTT	CGACGATTCCCAAAGAGAGA
MIP	TTTCAGCGAGCAGACAAACC	TCCCAGTGACGTGTTGGTAA

**Table 2:** Sequence of siRNAs.

Target Gene	Sense	Anti-Sense
Scrambled	AUGAACGUGAAUUGCUCAATT	UUGAGCAAUUCACGUUCAUTT
LPrx V	GCUAUAUACUCGUCGGUGGTT	CCACCGACGAGUAUAUAGCTT
SPrx V	GGAAGGAGACAGACUUAUUTT	AAUAAGUCUGUCUCCUUCCTT
MIP	GCCGGGAUCCGGGCCCGAATT	UUCGGGCCCGGAUCCCGGCTT

## Data Availability

Not applicable.

## Abbreviations

Prxs	peroxiredoxins
L-Prx V	long form Prx V
S-Prx V	short form Prx V
I-Prx V	intermediate Prx V containing octa peptide to be cleaved by MIP
Srx	sulfiredoxin
MTS	mitochondrial targeting sequence
MPP	mitochondrial processing peptidase
MIP	mitochondrial intermediate peptidase
ROS	reactive oxygen species
COX IV	cytochrome c oxidase subunit 4
GAPDH	glyceraldehyde-3-phosphate dehydrogenase
VDAC	voltage-dependent anion channels
ACTH	adrenocorticotropic hormone
Oct1	octapeptidyl aminopeptidase
